# Peer Review of “Raw, Unadulterated African Honey for Ulcer Healing in Leprosy: Protocol for the Honey Experiment on Leprosy Ulcer (HELP) Randomized Controlled Trial”

**DOI:** 10.2196/56498

**Published:** 2024-03-01

**Authors:** ­ Anonymous

**Keywords:** leprosy, ulcers, wounds, honey, neuropathy, nerves, Africa, randomized controlled trial, RCT

*This is the peer-review report for “Raw, Unadulterated African Honey for Ulcer Healing in Leprosy: Protocol for the Honey Experiment on Leprosy Ulcer (HELP) Randomized Controlled Trial.”*

## Round 1 Review

### General Comments

This is an excellent interventional protocol for a randomized controlled trial assessing honey as a potential ulcer therapeutic [1]. Careful consideration has been made to avoid bias and ensure robust results. I would suggest a few things to consider (below) prior to publishing.

### Specific Comments

#### Major Comments

##### Background and Rationale

- It is mentioned that 30% to 50% of people infected with leprosy have nerve damage. Be more specific here with “people”—is this a global estimate, American estimate, Nigerian estimate, etc?
- The background may benefit from a more specific discussion of previous literature. If there is a significant systematic review on the topic, a quick summary of relevant findings in the background (or later in the discussion) would help to situate the rationale behind carrying out such a study.

##### Study Setting

- St. Benedicts Tuberculosis and Rehab Hospital is owned by the Catholic Diocese. Do the authors suspect a potential religious bias in individuals who attend this hospital? Does this affect other social demographics and potentially skew generalizability?
- I am not sure it is necessary to go into this much detail about the staffing compositions and facilities of each site. Consider truncating.

##### Additional Consent Provisions

- This section mentions that the computer program will range-check information. Please specify which computer program.

##### Intervention Description

- The honey is being obtained from local bee farmers in North Central Nigeria; however, it is unclear how the honey is being prepared prior to inclusion into the study. I understand it is being checked for botulism (which is great); however—I wonder—is the honey from different farms being mixed together prior to use? Or is it possible that 1 dressing may be from 1 specific farm, etc? If so, is there a potential risk of interventional procurement bias? Meaning the honey from one farm may be better at wound healing then the honey from another farm? Just something to think about…
- Be more clear about the function of the video recording. Will it also be used to test if assessors can distinguish between honey versus control?

##### Confidentiality

- This section mentions that study forms containing personal identifier information will be kept secured and locked at trial site. Which trial site? Just 1? Or both? Please be more specific here.

##### Other

- Will you be collecting demographic data such as sex, gender, creed, socioeconomic status, level of schooling, etc? Would you consider stratifying results by any of these parameters?
- Additionally, will you be collecting information on leprosy status, that is, paucibacillary versus multibacillary leprosy or if the patient has progressed into the leprosy reaction stage (type 1 or type 2)? This information may be useful for downstream analysis and can be stratified for to avoid spectrum bias.

### Minor Comments

#### Background and Rationale

- Edit sentence to read: “…and peripheral nerves, causing neuropathy and severe disability, consequently resulting in social exclusion and stigmatization.”
- Edit sentence to read: “…new child cases [3], with a grade 2 disability rate of about 15% for the past…”
- Edit sentence to read: “Thirty to fifty percent of…”
- Edit sentence to read: “Ulcers usually occur in anesthetic feet, and will heal slowly with routine therapy, however have a tendency to recur [6].”
- Edit sentence to read: “…documented report record in the Edwin Smith Papyrus…”
- Edit sentence to read: ”…gathered and modified by the honeybee…”
- Edit sentence to read: “…exudates, and possesses antimicrobial…”
- Edit sentence to read: “…treatment of difference kinds of wounds, as researchers continue…”
- Edit sentence to read: “…sizeable number of reports that show mixed levels…”
- Edit sentence to read: “…with only about 5% of patients reporting pain following dressing.”
- The rest of the previous sentence “…and undocumented concern of botulism disease due to infection…” is unclear. Do the same 5% of patients also report concerns of botulism? Or is botulism a concern the authors have, and that has not been reported in previous literature? Either way I would make the botulism argument its own separate sentence that is more clear.

#### Study Setting

- Is it “St. Benedict’s TBL” or “St. Benedit’s TBL”? Please correct all instances to 1 or the other. The first sentence under study setting uses “St Benedits.”
- In “…is a TB and leprosy…” please type out “Tuberculosis” on first use with “(TB)” in quotes as per other abbreviations.

#### Eligibility Criteria

- Edit sentence to read: “…in the intervention group, all ulcers – not just the one…”
- Edit sentence to read: “Routine swabs will be taken, but the interpretation of…”

#### Additional Consent Provisions

- Edit sentence to read: “Photograph of the ulcers will be…”

#### Relevant Concomitant Care

- Edit sentence to read: “…bearing and the level of activity of patients might…”

#### Recruitment

- Edit sentence to read: “…identified by the on-site clinical…”

#### Plans for Assessment

- Edit sentence to read: “…database managers at the University of…”

#### Composition of the Data

- Edit sentence to read: “…Monitoring Committee consists of individuals…”
- Edit sentence to read: “…participant has been followed up for 84 days or discharged, whichever…”

#### Dissemination Plans

- Low- and middle-income countries needs to be fully written out at first use, then the abbreviation “LMIC” can follow.

#### Discussion

- Edit sentence to read: “…of its near absence from the global health agenda [25], and as such, very little…”
- Edit sentence to read: “A Cochrane review [31] noted that previously published evidence is limited, due to a high or unclear risk of bias (selection, performance, detection, or attrition) detected, imprecision due to little participants, indirectness due to poor outcome measures, and inapplicable interventions.”
- Edit sentence to read: “Although honey has been known for centuries to promoted wound healing, there are only a few controlled clinical trials that assess its efficacy.”
- This would be a good place to include a brief discussion of relevant findings with specific outcomes or statistics, as I mentioned in the background section.
